# Supplementary material for: Optimizing deep learning models for glaucoma screening with vision transformers for resource efficiency and the pie augmentation method
Source: PLoS One. 2025 Mar 21;20(3):e0314111. doi: 10.1371/journal.pone.0314111 (PMC11927916; doi:10.1371/journal.pone.0314111)
Supplement: Attachment — Submitted filename: res_reviews_new_2.pdf [file pone.0314111.s002.pdf]

April 17, 2024

PLOS ONE

Dear Reviewers,

We appreciate your email regarding our manuscript and the constructive feedback. We are committed to meeting PLOS ONE's submission requirements and have thoroughly reviewed your comments. We have addressed each point as detailed below:

> Thank you for your email regarding our manuscript and for providing constructive feedback. We are committed to addressing the issues outlined to ensure compliance with PLOS ONE's submission requirements. We have carefully reviewed your comments and suggestions and have addressed each one as outlined below:

1. Manuscript format:
   - I have taken note of the accepted file types for submission. Since my manuscript was prepared in LaTeX, I have attached the manuscript in PDF format and also included the .tex file, labeled as 'other'.

2. Data sharing restrictions:
   - There are ethical restrictions on publicly sharing the Chula—Thammasat Glaucoma dataset (GlauCUTU-DATA) used in this study. These restrictions are in place to protect the privacy of human research participants involved in the study. However, we can make a subset of the data available upon request. This subset includes:
     - Study ID: A unique identifier for each participant, ensuring anonymity.
     - Image shape: Details of the image dimensions
     - Data formats:
       (1) Fundus images with unanimous agreement by three random well-trained ophthalmologists (3/3).
       (2) Fundus images with majority agreement (2/3).
     - Test set: The subset is derived from the test set of the GlauCUTU-DATA, ensuring that the available data are representative of the conditions and findings presented in the study.
     - Groundtruth and Model Prediction: This subset includes details of the ground truth and the model's predictions to enable independent verification of the accuracy metrics reported in our manuscript. Within this dataset, 'G' represents glaucoma, 'S' represents glaucoma suspect, and 'N' represents no glaucoma.

   - The data used in this study contains sensitive patient information and is owned by a third-party organization, King Chulalongkorn Memorial Hospital, Thai Red Cross Society, Bangkok, Thailand. As a result, the Institutional Review Board (IRB) of the Faculty of Medicine, Chulalongkorn University, Bangkok, Thailand has imposed restrictions on public data sharing. Researchers interested in accessing this dataset can contact the IRB human research office at King Chulalongkorn Memorial Hospital, Thai Red Cross Society using the following information:
     - Contact: IRB Office, 3rd Floor, Anandamahidol Building
     - Telephone: 0 2256 4493 / 098 573 7622
     - Email: medchulairb@chula.ac.th
     - Website: https://irb.md.chula.ac.th

Thank you for your guidance and consideration of our work. We look forward to your response and are eager to proceed with the publication process.

Sincerely,

Rath Itthipanichpong (corresponding author)
E-mail: doctorrath@gmail.com
